# Atomic layer deposition of rhodium and palladium thin film using low-concentration ozone†

**DOI:** 10.1039/d1ra03942c

**Published:** 2021-06-28

**Authors:** Yiming Zou, Chunyu Cheng, Yuanyuan Guo, Amanda Jiamin Ong, Ronn Goei, Shuzhou Li, Alfred Iing Yoong Tok

**Affiliations:** School of Materials Science and Engineering, Nanyang Technological University Singapore 639798 Singapore MIYTok@ntu.edu.sg lisz@ntu.edu.sg

## Abstract

Rhodium (Rh) and palladium (Pd) thin films have been fabricated using an atomic layer deposition (ALD) process using Rh(acac)_3_ and Pd(hfac)_2_ as the respective precursors and using short-pulse low-concentration ozone as the co-reactant. This method of fabrication does away with the need for combustible reactants such as hydrogen or oxygen, either as a precursor or as an annealing agent. All previous studies using only ozone could not yield metallic films, and required post treatment using hydrogen or oxygen. In this work, it was discovered that the concentration level of ozone used in the ALD process was critical in determining whether the pure metal film was formed, and whether the metal film was oxidized. By controlling the ozone concentration under a critical limit, the fabrication of these noble metal films was successful. Rhodium thin films were deposited between 200 and 220 °C, whereas palladium thin films were deposited between 180 and 220 °C. A precisely controlled low ozone concentration of 1.22 g m^−3^ was applied to prevent the oxidation of the noble metallic film, and to ensure fast growth rates of 0.42 Å per cycle for Rh, and 0.22 Å per cycle for Pd. When low-concentration ozone was applied to react with ligand, no excess ozone was available to oxidize the metal products. The surfaces of deposited films obtained the RMS roughness values of 0.30 nm for Rh and 0.13 nm for Pd films. The resistivities of 18 nm Rh and 22 nm Pd thin films were 17 μΩ cm and 63 μΩ cm.

## Abstract 2

Rh and Pd metallic thin films were fabricated by atomic layer deposition using Rh(acac)_3_ and Pd(hfac)_2_ precursors, and only low-concentration ozone as co-reactant.

## Graphical abstract

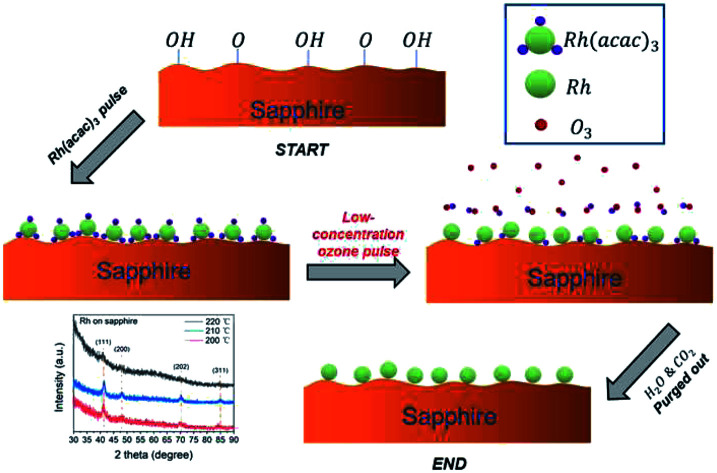

## Introduction

Noble metals like rhodium (Rh) and palladium (Pd) have drawn academic attention since their properties were realized. Rh is widely used as a catalyst for the hydrogenation of carbon dioxide,^1^ hydrogen production^2,3^ and styrene production from benzene and ethylene,^4^*etc.* Rhodium nanoparticles and clusters have also been widely used in gas detection and sensing.^5–8^ Similarly, Pd is also well-known as a catalyst for oxidation of carbon monoxide and organic compounds.^9,10^ In addition, Pd is also an important material in fields of hydrogen sensing^11–14^ and hydrogen storage.^15–19^ For all the unique properties mentioned above, a larger surface area is required to promote the properties of these noble metals. To achieve this purpose, the coated thin film of nanoparticles with large specific surface area on the substrates or the surface of 3D surface is expected. Since the specific surface area is determined by the nanoparticle size, a controllable nanoparticle size is desirable. Compared with thin film, a bulk material fabricated from noble metals would be less efficient and extremely costly. Besides nanoparticle size, the distribution of it is also a critical factor that influences the performance of the materials. Atomic layer deposition (ALD) has been proven to be a promising method that allows the precise, conformal Rh and Pd deposition with controllable nanoparticle size and size distribution.^20–22^

Compared with other noble metal ALD processes, Rh ALD is limited, as only rhodium(iii) acetylacetonate (Rh(acac)_3_) has been found to be a feasible precursor. In 2005, Aaltonen *et al.* reported Rh ALD process using Rh(acac)_3_ as the precursor and oxygen as the co-reactant.^23^ Another study further proved the feasibility of this process, whose result was consistent with the previous study.^24^ The use of Rh(acac)_3_ precursor coordinating with ozone and lower deposition temperature only resulted in Rh_2_O_3_ thin films. Partial reduction of Rh_2_O_3_ to Rh occurred at 190 °C,^25^ but no further study has obtained pure Rh thin films by continuing increasing the deposition temperature or trying lower ozone concentration. In a later study, H_2_ pulse was introduced after each ozone pulse to reduce the oxide products to obtain the metallic Rh thin films.^26^ However, as an explosive and flammable gas, H_2_ is so dangerous that it is prohibited by many institutions and prevented from large-scale preparation in industry.

Among noble metal ALD processes using metal acetylacetonate (acac) species as the precursors, Pt(acac)_2_ (ref. 27) and Ir(acac)_3_ (ref. 28) could both achieve Pt and Ir metallic films respectively when ozone was used as the co-reactant. For both Pt and Ir deposition, metal oxide thin film was obtained at low temperature, while metallic thin film was obtained at higher temperature. A threshold temperature was defined as the point where the metallic film started to form in place of the oxide film. Above the threshold temperature, the available oxygen was quickly consumed by the ligand and as such prevented the oxidation of Pt or Ir, leaving the metallic surface. Since Rh belongs to platinum group metals and the molecular structure of Rh(acac)_3_ is very similar to that of Ir(acac)_3_, it is expected that there is also a threshold temperature for Rh(acac)_3_–O_3_ process, above which pure metallic films will be obtained. Besides temperature, the concentration of oxidizing gas is also an important factor that influences noble metal ALD. It was demonstrated that the concentration of oxygen during the ALD process directly determined the products of reaction between Ru(EtCp)_2_ and ozone.^29^ Under high concentration ozone, oxygen could easily diffuse into Ru lattice.^30^ In previous Rh(acac)_3_–O_3_ process, 100 g m^−3^ high concentration ozone was applied, and only the oxide product was obtained.^25^ As such, it is postulated that Rh thin film could similarly be achieved at higher temperature with precisely controlled low-concentration ozone.

The same process with low concentration ozone was also applied to the Pd ALD process with palladium(ii) hexafluoroacetylacetonate (Pd(hfac)_2_) precursor. Pd(hfac)_2_ is the most widely used precursor in Pd ALD. Depositions of Pd thin film coordinated with H_2_ (ref. 31 and ^32^) and formalin^33,34^ on Al_2_O_3_ have been proven feasible. However, in those studies, the growth of Pd thin film was hampered by Al(hfac) surface species, leading to a longer nucleation period and growth delay at the beginning of the ALD process.^35^ Weber *et al.* attributed the problem of Al(hfac) to the carbon contamination and promoted the process by using another O_2_ plasma after each H_2_ plasma to eliminate the contamination.^36^ As a result, Pd thin films with high uniformity and purity were achieved with Pd(hfac)_2_. Feng *et al.* further compared the cycle sequences of Pd(hfac)_2_–H_2_, Pd(hfac)_2_–H_2_–O_2_, Pd(hfac)_2_–O_2_–H_2_ and Pd(hfac)_2_–NH_3_ in one study.^37^ However, all these known methods require the use of H_2_ as a reactant or as an annealing agent. To date, all reported attempts to fabricate Pd thin film with Pd(hfac)_2_ and another oxidizing agent, such as H_2_O_2_, H_2_O, O_2_ and ozone^38^ have failed in achieving pure metallic films.

In this study, Rh and Pd thin films were fabricated by using Rh(acac)_3_, Pd(hfac)_2_ as the precursors and precisely controlled low-concentration ozone as the co-reactant. To figure out magnitude of ozone concentration, the calculation was carried out based the assumption that all the C, H in the precursors reacted with ozone to form CO_2_ and H_2_O. In this case, the chemical equations were written as:

For Rh(acac)_3_,

1 2Rh(O_2_C_5_H_7_)_3_(ads) + 23O_3_(g) = 2Rh(s) + 30CO_2_(g) + 21H_2_O(g).

For Pd(hfac)_2_,

2 3Pd(C_5_HF_6_O_2_)_2_(ads) + 17O_3_(g) = 3Pd(s) + 30CO_2_(g) + 3H_2_O(g) + 36F(ads).

The calculated ozone concentration was 2.16 g m^−3^ for Rh ALD and 1.92 g m^−3^ for Pd ALD, respectively. During the experiment, different ozone concentration was tested and 1.22 g m^−3^ was finally selected as the ozone concentration.

All thin films were deposited on sapphire substrates, obtaining low resistivity and uniform surface. The growth of Rh revealed an obvious delay, whereas the growth of Pd was linear from the beginning. The morphology deformation of Pd thin films with deposition temperature was discovered.

## Simulation details

The first-principle calculations based on DFT were used in this work to reveal the reaction mechanism between Pd(hfac)_2_ and ozone. The projector-augmented wave (PAW) pseudopotential method^39,40^ and generalized gradient approximation (GGA) – Perdew–Wang 1991 (PW91) functional^41^ were executed by Vienna *Ab Initio* Simulation Package (VASP).^42,43^ The simulation parameters and models are described in ESI.†

## Experimental section

Rhodium(iii) acetylacetonate (Rh(acac)_3_, 97%) was purchased from Sigma Aldrich. Palladium(ii) hexafluoroacetylacetonate (Pd(hfac)_2_, min, 95%) was purchased from Strem Chemicals. High purity nitrogen (99.999%) was used as the carrier and purging gas. Ozone was obtained by converting high purity oxygen (99.999%) with the ozone generator (Nanofrontier, XLK-G20). 1 × 1 cm^2^ sapphire (α-Al_2_O_3_(0001)) was selected as the substrates.

All experiments have been carried out in a custom-built ALD reactor. 150 sccm nitrogen flow continuously passed through the main chamber to keep a base pressure of 1.0 mbar. The sapphire substrates were washed in deionized water, 95% ethanol (with 5% methanol), acetone and isopropyl alcohol in ultrasonic bath respectively. Each procedure lasted for 5 min. Substrates for Rh fabrication were then kept at 500 °C for 4 h in a tube furnace to create reactive sites for Rh nucleation,^44^ whereas others were blown dry with high purity nitrogen flow and stored for Pd fabrication. Rh(acac)_3_ was loaded in a stainless-steel bubbler (Strem Chemicals, 150 mL), while Pd(hfac)_2_ was loaded in a glass bubbler. The sublimation temperature for Rh(acac)_3_ and Pd(hfac)_2_ was set as 165 °C and 65 °C respectively, to obtain enough vapor pressure. The transfer tube between the bubbler and main chamber was permanently kept at 175 °C and 100 °C for Rh(acac)_3_ and Pd(hfac)_2_ respectively to prevent the condensation of the precursors.

The main process consisted of four main periods: pulse of carrier gas into the precursor holder (*t*_1_), pulse of precursor (*t*_2_), pulse of ozone (*t*_3_) and purging time (*t*_4_). Then all the processes were set as *t*_1_–*t*_2_–*t*_4_–*t*_1_–*t*_2_–*t*_4_–*t*_3_–*t*_4_, and the specific duration of each period is listed in Table 1. In both processes, considering the vapor pressures of the precursors were limited, ozone was pulsed after two repetitive precursor pulses so that the precursors can fully attach to the surface.

**Table 1:** Pulse details for the Rh and Pd ALD processes

	*t*_1_ (ms)	*t*_2_ (ms)	*t*_3_ (ms)	*t*_4_ (ms)
Rh(acac)_3_	5000	2000	300	2000
Pd(hfac)_2_	500	500	100	2000

The microstructure of the films was studied using grazing incidence X-ray diffraction (Bruker D8 discover XRD System, GI-XRD) with high intensity (*λ*_Cu-Kα_ = 1.54 Å) radiation and a grazing angle of 1°. The scan speed of 0.5 seconds and 0.01° per step was applied. The thickness and density of the films were measured by X-ray reflectometry (XRR) on the same diffractometer. The elemental and binding information was characterized using X-ray photoelectron spectroscopy (Shimadzu Kratos Axis Supra, XPS) with an Al K_α_ source. The surface morphology was characterized with field emission scanning electron microscopy (JEOL, 7600F, FESEM) and atomic force microscopy (Park System NX10, AFM). The RMS roughness was determined over a 1 × 1 μm^2^ area by the same AFM.

## Results and discussion

### Simulation results

The reaction mechanisms of direct palladium synthesis using Pd(hfac)_2_ and ozone on substrate surface by ALD are investigated using DFT simulations. The ALD process starts by the dissociative chemisorption of Pd(hfac)_2_ to form the adsorbed Pd(hfac) and hfac (cited as “Pd(hfac)*” and “hfac*” respectively) on substrate surface. As an ozone molecule approaches to Pd(hfac)*, an adsorbed Pd atom (cited as “Pd*”) can be produced by an O atom of the ozone at the bridging C–C site of Pd(hfac)* where the C–C bond is cleaved with an activation barrier of 0.46 eV. The O atom of the Pd–O–C bonds of Pd(hfac)* can be also replaced by an O atom of the ozone at the top Pd site to form the adsorbed Pd(hfac–O) (cited as “Pd(hfac–O)*”) with a lower activation barrier of 0.29 eV. When more ozone molecules are available, an activation barrier of 0.42 eV needs to be overcome for Pd* formation by an O atom of the ozone at the bridging C–C site of Pd(hfac–O)* to cleave the C–C bond. The ozone molecule at the top Pd site can also bond with the Pd atom of Pd(hfac–O)* to form the adsorbed O_3_–Pd(hfac–O) (cited as “O_3_–Pd(hfac–O)*”), which is reversible and barrierless. The O_3_–Pd(hfac–O)* then decomposes into the adsorbed O–Pd(hfac–O) and gaseous oxygen with a high activation barrier of 0.83 eV, meaning that PdO formation is kinetically less favourable. Besides, the formed Pd* can be oxidized by the ozone at the Pd–Si site with an activation barrier of 0.64 eV. Based on activation barriers from DFT calculations, it is possible to prepare palladium without reducing steps when ALD conditions are carefully controlled, especially the control of the ozone concentration. The simulation details are described in ESI.†

### Rhodium thin films

Pure metallic Rh films were obtained at 200, 210 and 220 °C, while no film was found at 190 °C. Combining the results of reported Rh(acac)_3_–O_3_ processes (shown in Table 2), the threshold temperature should be between 190 and 200 °C. Apart from temperature, the ozone concentration also has influence on the products of the ALD process. With lower ozone concentration, the deposition temperature increased, while the oxide product was less likely to form.

**Table 2:** The temperature, ozone concentration and products of previous studies and this work

Cycle sequence	Temperature	Ozone concentration	Products	Ref.
Rh(acac)_3_–O_3_	160–180 °C	100 g m^−3^	Rh_2_O_3_	25
Rh(acac)_3_–O_3_	190 °C	100 g m^−3^	Rh_2_O_3_ + Rh	25 and 26
Rh(acac)_3_–O_3_	190 °C	1.22 g m^−3^	No film	This work
Rh(acac)_3_–O_3_	200–220 °C	1.22 g m^−3^	Rh	This work

**Fig. 1:**
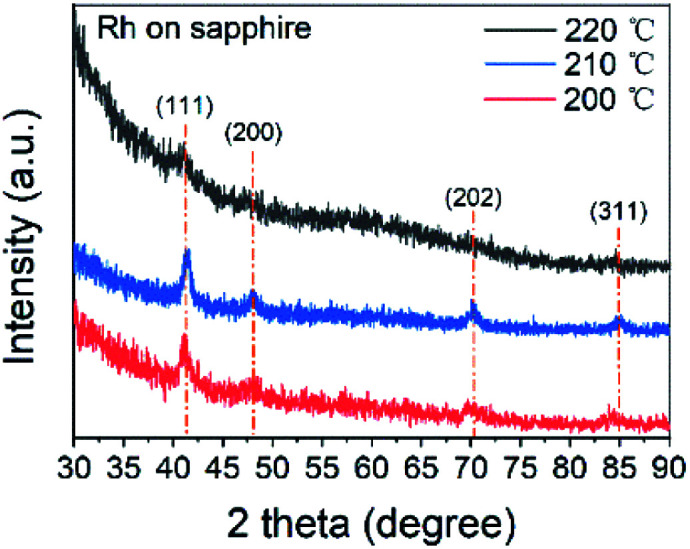
The XRD patterns of Rh thin films deposited at 200, 210 and 220 °C on sapphire substrates.

GI-XRD measurement with 1° grazing angle was carried out to study the microstructure of the thin films. The XRD spectra in Fig. 1 were obtained from 18 nm Rh thin films deposited on sapphire at 200, 210 and 220 °C. The peaks of (111) (2*θ* = 40.77°), (200) (47.44°), (202) (59.34°) and (311) (83.68°) revealed the preferred (111) orientation of face-centred cubic Rh. From the full width at half maximum (FWHM) of peak (111), the grain size was determined as 10 ± 1 nm by Debye Scherrer equation. Compared with spectrum at 200 °C, spectrum at 210 °C showed higher intensity, revealing higher crystallinity. However, at 220 °C, the peaks were so blurred that only the peak of (111) can be clearly recognized, implying amorphous Rh or Rh_2_O_3_. Since the resistivity of thin films deposited at 220 °C was only 17 μΩ cm, it should be assigned to pure metal. Conversely, the resistivity of Rh_2_O_3_ should exceed 6000 μΩ cm.^25^ Therefore, it was believed that thin films deposited at 220 °C were amorphous Rh. The resistivity of the thin films deposited at 200, 210 and 220 °C was 17 ± 5 μΩ cm, which was slightly higher than those in previous Rh ALD studies.^23^ The increase in resistivity of noble metal thin films when thickness was less than 20 nm was reported to be caused by the electron-surface and electron-boundary scattering when the electron mean-free-path is close to the grain size and the thin film thickness.^45,46^

**Fig. 2:**
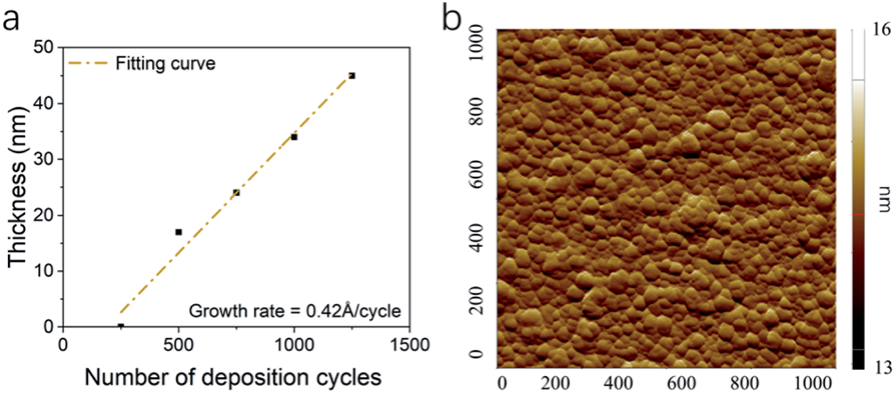
The (a) thickness of Rh thin films with number of deposition cycles and (b) AFM image of Rh thin film surface 1 × 1 μm^2^ area.

The thicknesses of thin films were determined by XRR. Thin films with 250 cycles shown very limited coverage of the surface. This result indicated a nucleation process and delay of growth during the Rh ALD process. After the nucleation, Rh thin film shown a linear growth. The growth rate of 0.42 Å per cycle was determined by fitting the thickness data (Fig. 2a), which is slower than those in previous studies. The density of Rh thin films was 5.6 g cm^−3^, lower than the density of Rh bulk. For Rh thin films deposited on sapphire, the RMS roughness was 0.30 nm (Fig. 2b). Compared with Rh deposition through Rh(acac)_3_–O_3_–H_2_ process,^36^ where the surface roughness was 0.9 nm for 23 nm Rh thin film, the surface in this study is much smoother. Many factors could contribute to the low roughness, including more uniform ozone flux,^25^ less cycle number,^47^ more uniform temperature and its distribution in reactor,^48^*etc.*

**Fig. 3:**
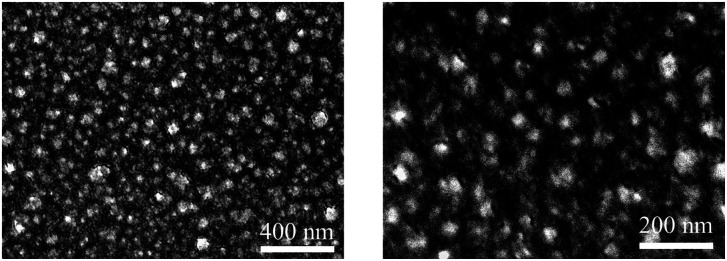
FE-SEM images of 18 nm Rh thin films deposited on sapphire at 210 °C at different magnitudes. 500 deposition cycles were applied.

The morphology of Rh thin film surface was studied through SEM images (Fig. 3). The images of Rh deposited at 210 °C with thickness of 18 nm are shown in Fig. 3 Island-shape Rh nanoparticles were uniformly arranged on the substrate. The diameter of the nanoparticles is 40 ± 8 nm. The morphology and particle size were similar to that of 25 nm Rh thin films deposited by using Rh(acac)_3_ and oxygen as the co-reactants.^23^ It was generally believed that the particle size increases with increasing cycle number applied in the ALD process. More recent studies also revealed that the pressure of gas phase reactant also affected the particle size and the distribution: the lower the pressure, the larger the average diameter of nanoparticles and wider nanoparticle size distribution.^49^ From this conclusion, in Fig. 3, the morphology resulted from low concentration ozone could be explained: although the surface is uniformly occupied by nanoparticles, their distribution was relatively wide.

**Fig. 4:**
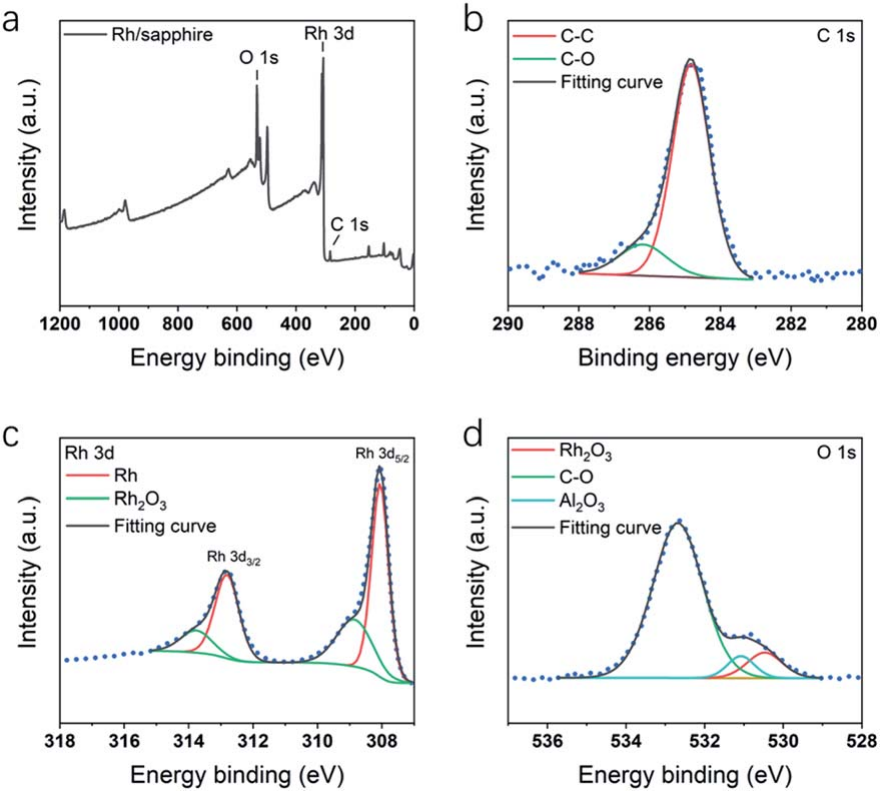
XPS spectra of Rh thin films deposited on sapphire at 210 °C, including spectra of (a) survey signal, (b) C 1s, (c) Rh 3d and (d) O 1s.

Fig. 4 shows the XPS survey spectrum and spectra of the C 1s, Rh 3d and O 1s core-levels obtained from Rh thin film deposited at 210 °C. All the spectra have been calibrated using C–C peak at 284.8 eV (Fig. 4b). In Rh 3d spectrum (Fig. 4c), the major components at 308.1 eV and 312.8 eV (*Δ* = 4.7 eV) were assigned to metallic Rh_5/2_ and Rh_3/2_. Another two minor components at 308.9 and 313.7 eV were assigned to Rh_2_O_3_. The existence of oxide component was also verified by the component at 530.4 eV in O 1s spectrum (Fig. 4d). According to the XPS study of Rh,^50,51^ the components were identified as Rh_2_O_3_. The oxidation could probably result from adsorption of oxygen molecules when exposed to the air. In O 1s spectrum in Fig. 4c, except for Rh_2_O_3_ component, a major C–O component at 532.6 eV indicated some remained ligand from Rh(acac)_3_.

### Palladium thin films

**Fig. 5:**
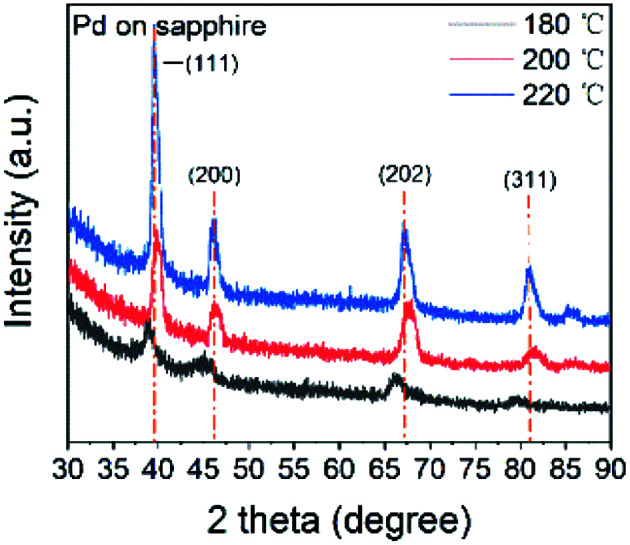
The XRD patterns of Pd thin films deposited on sapphire at 180, 200 and 220 °C.

All the thin films were determined as metallic palladium by XRD patterns of 22 nm Pd thin films on sapphire deposited at 180, 200 and 220 °C (Fig. 5). The peaks of (111) (2*θ* = 39.92°), (200) (46.43°), (202) (67.77°) and (311) (81.65°) verified the face-centered cubic Pd. The average grain size was determined as 11.6 nm. As the temperature increased, the intensity of specialized peaks was enhanced, indicating the higher crystallinity at higher deposition temperature. Furthermore, compared with the patterns of the thin film deposited at 200 and 220 °C, the pattern at 180 °C shows a shift to lower 2*θ* value, implying an isotropic expansion of the lattice. The calculated unit cell dimension is 0.401 nm for 180 °C, larger than 0.391 nm for 200 and 220 °C. The peak shift can be attributed to carbon incorporation in Pd lattice.^36,52,53^ In this case, the elimination of carbon contamination occurred when the deposition temperature continued increasing. The resistivity of Pd thin films deposited at 180, 200 and 220 °C was 63 ± 5 μΩ cm, which was much higher than 9.8 μΩ cm of pure bulk palladium. Since the electron mean free path of palladium is 25.5 nm,^54^ which is even longer than the calculated diameter of Pd grain, the electron-grain boundary scattering would be greatly enhanced. Moreover, according to the previous Pd ALD study in which the resistivity was 48 μΩ cm,^36^ the increase in resistivity was also attributed to the carbon content.

**Fig. 6:**
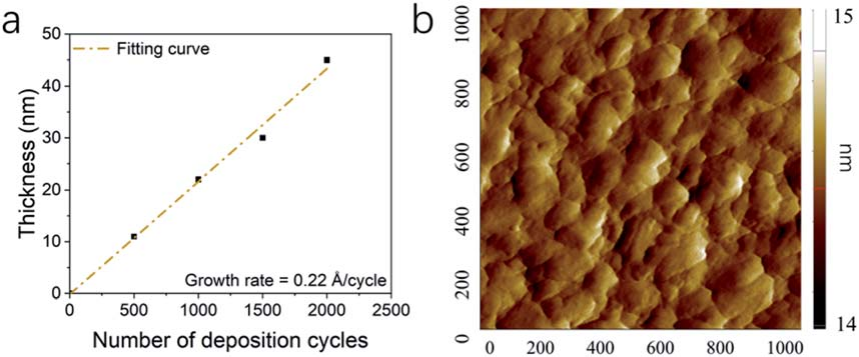
The (a) thickness of Pd thin films with number of deposition cycles and (b) AFM image of Pd thin film surface over 1 × 1 μm^2^ area.

**Fig. 7:**
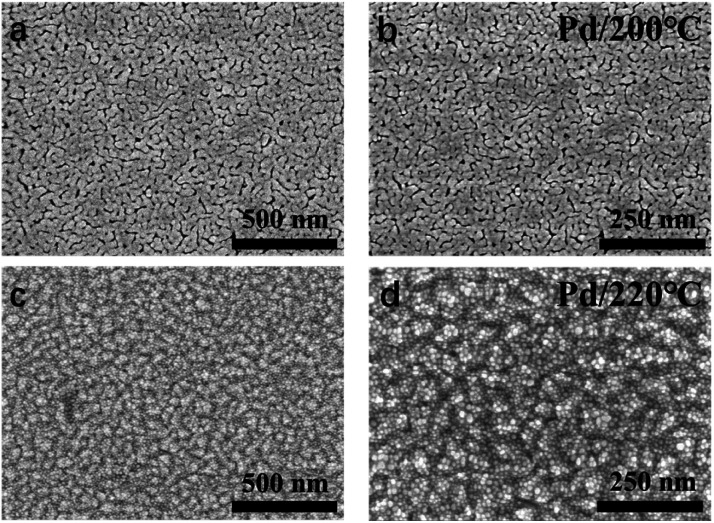
FE-SEM images of 22 nm Pd thin films deposited on sapphire at 200 °C (a) and (b) and 220 °C (c) and (d) 1000 deposition cycles were applied for both temperatures.

The growth of Pd thin film was different from Rh deposition, as the growth shown in Fig. 6a showed no obvious delay. The growth rate was determined as 0.22 Å per cycle. The AFM image is shown and Fig. 6b and the RMS roughness was determined as 0.13 nm, which was smoother than the roughness reported in previous studies (Table 3). The SEM images of Pd thin films at 200 and 220 °C in Fig. 7 revealed a change in morphology with increasing temperature. For the thin film deposited at 200 °C, the morphology showed the highly coalesced structure. But when the deposition temperature was increased to 220 °C (Fig. 7c and d), the morphology became island-shape nanoparticles. The nanoparticles with size of 7 ± 3 nm were uniformly distributed on the surface.

**Table 3:** The thickness and roughness of palladium thin films reported in previous studies

Cycle sequence	Area (μm^2^**)**	Thickness (nm)	Roughness (nm)	Ref.
Pd(hfac)_2_–H_2_ plasma	0.5 × 0.5	2	2.1	31
Pd(hfac)_2_–H_2_ plasma	0.5 × 0.5	4	0.4	31
Pd(hfac)_2_–H_2_	0.5 × 0.5	3	0.5	32
Pd(hfac)_2_–formalin	1 × 1	42	4.2	33
Pd(hfac)_2_–H_2_ plasma	2 × 2 μm^2^	17	1.0	36
Pd(hfac)_2_–H_2_ plasma–O_2_ plasma	2 × 2 μm^2^	20	1.3	36
Pd(hfac)_2_–formalin		25	3.5	34
Pd(hfac)_2_–O_3_	1 × 1 μm^2^	20	0.13	This work

The change in morphology contradicted the general understanding of the aggregative growth of ALD. It is generally believed that lower temperature results in smaller particle size and narrower particle size distribution, as carbon on the surface prevents nanoparticles from coalescence by suppressing their mobility.^46,55,56^ However, in this work, Pd nanoparticles that grew at 200 °C were highly coalesced, while at 220 °C were well-separated. The island coalescence was also reported in other Pt and Pd ALD studies.^33,57^ It was attributed to the poor nucleation. When the pulses of trimethylaluminum (TMA) were applied prior to the main processes, more nucleation sites were created on the surface, and the nanoparticles became well-separated.^58,59^ Therefore, in this work, the change in morphology of Pd thin films with increasing temperature indicated an enhancement of nucleation.

Combining with XRD result mentioned above, we proposed that the decomposition of (hfac) species (the poisoning species) at 220 °C enhanced the nucleation of Pd. It has been proved that CF_3_ species partly decomposed when the temperature went beyond 200 °C, and (hfac) species full decomposed at 252 °C.^35,60^ At 220 °C, the surface could be occupied with CF_2_, C, F and Pd atoms, rather than (hfac) species. During the ALD process, fluorine was reacted away, and the surface carbon help fix and separate the nanoparticles.

**Fig. 8:**
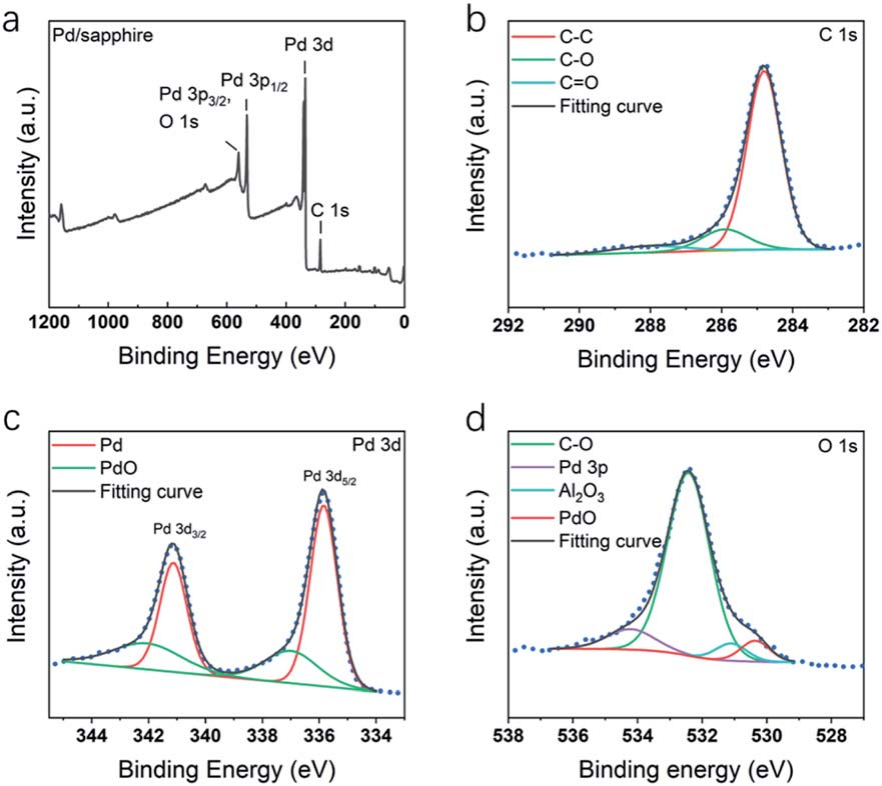
XPS spectra of Pd thin films deposited on sapphire at 200 °C, including spectra of (a) survey signal, (b) C 1s, (c) Pd 3d and (d) O 1s.

Fig. 8 shows XPS spectra of the C 1s, F 1s, O 1s, and Pd 3d core-levels obtained from Pd thin film deposited at 200 °C. In C 1s spectrum, except C–C component, C–O peak at 285.9 eV and C=O peak at 288.0 eV were identified. As no C–F was detected, it was indicated that fluorine element has been eliminated during the ALD process. Pd 3d spectrum was shown in Fig. 8c, the major component at 335.8 eV and its spin–orbit component (*Δ* = 5.26 eV) at 341.1 eV were assigned to metallic Pd_5/2_ and Pd_3/2_. Another two minor components at 337.1 and 342.2 eV were assigned to the oxide components. In O 1s spectrum (Fig. 8d), the peaks of C–O, Al_2_O_3_, PdO and Pd 3p were highly overlapped. Each component was identified by comparing the XPS spectra of the Pd thin film after fully oxidizing the thin film.

## Conclusions

In this study, Rh and Pd thin films were obtained by ALD method using Rh(acac)_3_ and Pd(hfac)_2_ as the precursors, ozone as the co-reactant. The short pulses of low-concentration ozone and the appropriate deposition temperature achieved metallic films with smooth surface, controllable thickness and fast growth rate. For Rh deposition, above the threshold temperature, low concentration ozone was consumed fast by the ligands, leading to the pure metallic surface. For Pd deposition, the metallic films were deposited within the temperature range of 180 to 220 °C. In addition, the enhancement of nucleation was discovered at 220 °C, when (hfac) species partly decomposed. Both deposition temperature and ozone concentration were critical to the formation of high purity metallic Rh and Pd thin films. With this ALD process, only a small amount of ozone joined the process, and no dangerous hydrogen would be used to reduce the metal oxides.

## Author contributions

A. I. Y. Tok conceived the project. Y. M. Zou and C. Y. Cheng co-wrote this paper. Y. M. Zou and Y. Y. Guo designed the experiments, performed ALD processes, and characterization. C. Y. Cheng accomplished simulation work. A. J. M. Ong corrected the English. R. Goei performed XPS and SEM characterization. All authors discussed the results, finalized the conclusions, and contributed to the manuscript.

## Conflicts of interest

There are no conflicts to declare.

## Supplementary Material

RA-011-D1RA03942C-s001
