# Supplementary material for: Atomic layer deposition of rhodium and palladium thin film using low-concentration ozone
Source: RSC Adv. 2021 Jun 28;11(37):22773–9. doi: 10.1039/d1ra03942c (PMC9034295; doi:10.1039/d1ra03942c)
Supplement: RA-011-D1RA03942C-s001 [file RA-011-D1RA03942C-s001.pdf]

Electronic Supplementary Material (ESI) for RSC Advances.
This journal is © The Royal Society of Chemistry 2021

Atomic Layer Deposition of Rhodium and Palladium Thin Film using Low-Concentration Ozone

Yiming Zou, ‡ Chunyu Cheng, ‡ Yuanyuan Guo, , Amanda Jiamin Ong, Ronn Goei, Shuzhou Li *, Alfred Iing Yoong Tok *

‡Co-first author

School of Materials Science and Engineering, Nanyang Technological University, Singapore 639798, Singapore. Email: MIYTok@ntu.edu.sg; lisz@ntu.edu.sg.

Supporting Information

1. XPS spectra of Rh & Pd thin film

As the intensities of oxide components in both Rh 3d and O 1s spectra were low and overlapped with metallic Rh and Al_2O_3 peaks, it was difficult to identify the components. To verify the assignments, the Rh thin film was kept in a tube furnace at 800 ℃ for 4h in air to get fully oxidized. [1] After heat treatment, the resistivity of the thin film increased to 17000 μΩ cm. The Rh 3d and O 1s spectra after heat treatment are shown in Fig. S1(d, e). The peaks of Rh were absent. Instead, the intensities of oxide components became stronger, while the positions of them do not change.

The fluorine elimination was also verified by F 1s spectrum in Fig. S2c, in which no component was discovered. However, in O 1s spectrum in Fig. S2e, it was difficult to identify the oxide component due to the overlapping of C-O, Pd-O and Pd 3p signals. Same as Rh XPS, to fully understand all the components, the Pd thin film was sent for heat treatment at 800 ℃ for 4h in air to get fully oxidized. The Pd 3d and O 1s spectra after the heat treatment were shown in Fig. S2(f, g). The Pd peaks disappeared and the asymmetric pairs at 337.4 eV and 342.6 eV were assigned to PdO. In O 1s spectrum, the three components were successfully separated. The peaks of PdO at 530.4 eV, C-O peak at 532.2 eV, Pd 3p peak at 534.3 eV were identified.

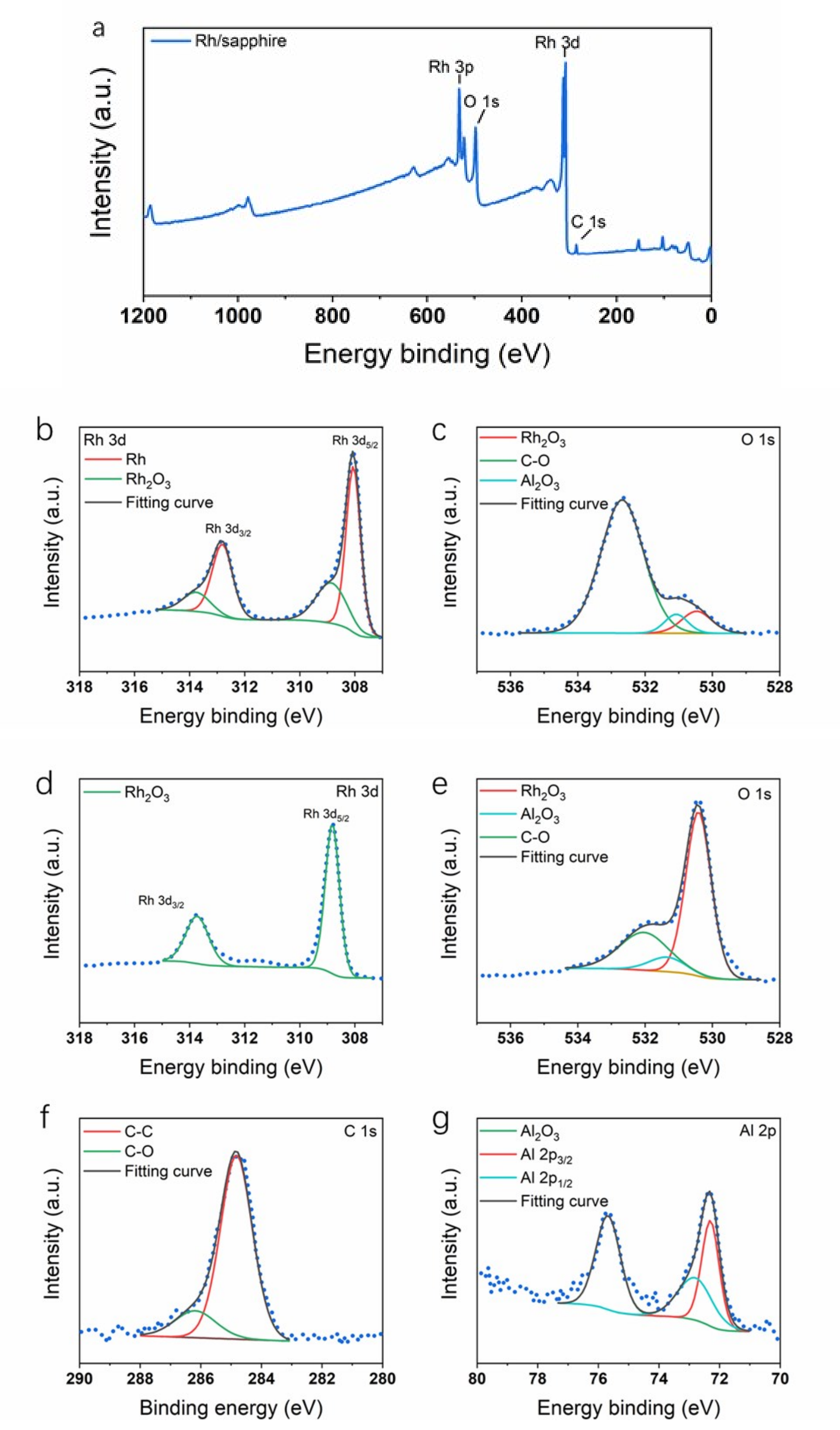

Fig. S1 XPS spectra of Rh thin films deposited on sapphire at 210 ℃, including spectra of **(a)** survey signal, **(b)** Rh 3d, **(c)** O 1s, **(d)** Rh 3d after heat treatment, **(e)** O 1s after heat treatment, **(f)** C 1s and **(g)** Al 2p. Spectra of Rh 3d and O 1s before and after heat treatment were compared to help identify the components.

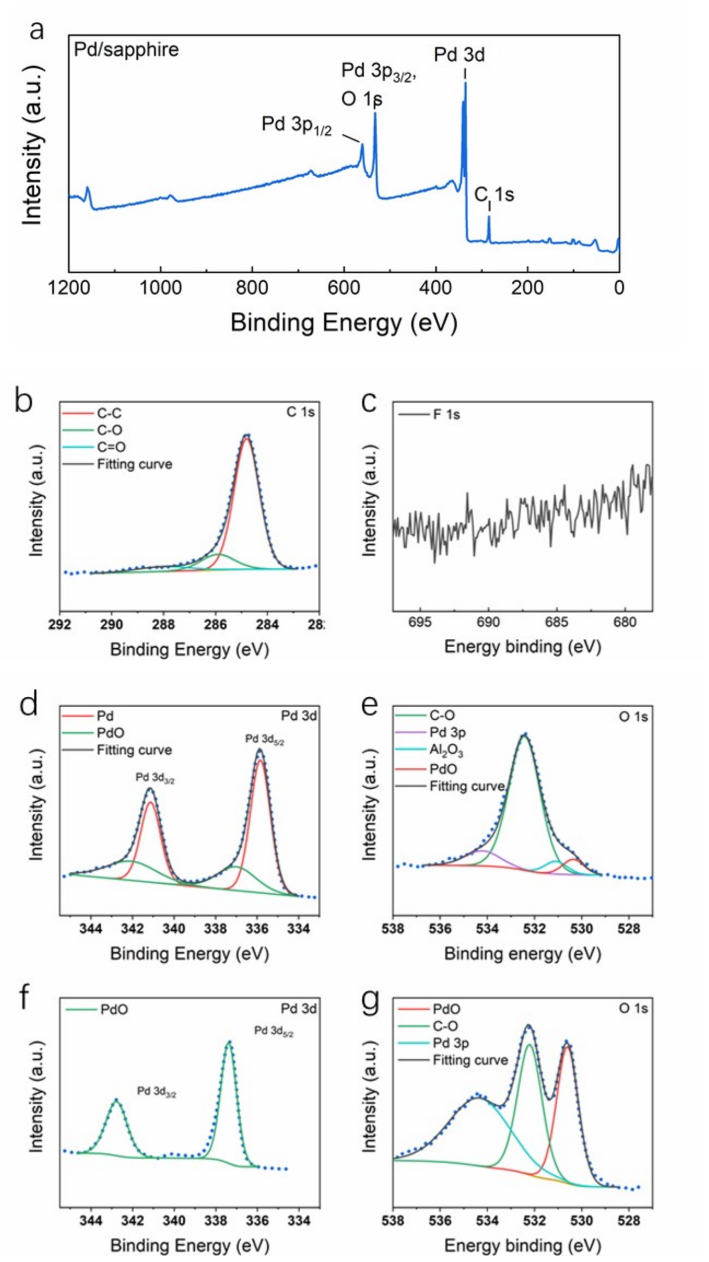

Fig. S2 XPS spectra of Pd thin films deposited on sapphire at 200 ℃, including spectra of **(a)** survey signal, **(b)** C 1s, **(c)** F 1s **(d)** Pd 3d, **(e)** O 1s, **(f)** Rh 3d after heat treatment, **(g)** O 1s after heat treatment. Spectra of Rh 3d and O 1s before and after heat treatment were compared to help identify the components.

2. DFT simulation of the reaction between $Pd(hfac)_2$ and ozone

2.1 Methodology

Up to 400eV of energy cutoff was employed for the plane-wave basis set, enough for the total energy. The absolute value of force on each unconstrained atom <0.001 eV/Å was carried out for geometry optimization. Besides, the Brillouin zone was sampled using a Monkhorst-Pack k-point mesh of 2 × 2 × 1.[1] The transition-state structure was obtained using the nudged-elastic-band (NEB) method[2,3] to simulate the minimum-energy profile along the reaction pathway connecting the reactant and product chosen based on the optimized structure.

A dissociation process for the adsorbed $Pd(hfac)_2$ (cited as "$Pd(hfac)_2$*") (reaction (S1)) was considered in this work to achieve a basic intuition,[4] even though the actual dissociation might be more complicated.

The reaction energy ΔE_{re1} for reaction (S1) was written as equation (S2).

$$\mathrm{Pd(hfac)_2}^* + {}^* \rightarrow \mathrm{Pd(hfac)}^* + \mathrm{hfac}^* \quad \text{(S1)}$$

Where Pd(hfac)* and hfac* represent the adsorbed Pd(hfac) and hfac respectively. The asterisk symbol means open surface, as defined below for $E_{\mathrm{surf}acce}$.

$$\Delta E_{\mathrm{re1}} = E_{\mathrm{Pd(hfac)}^*} + E_{\mathrm{hfac}^*} - E_{\mathrm{surf}ace} - E_{\mathrm{Pd(hfac)_2}^*} \quad \text{(S2)}$$

Where $E_{\mathrm{Pd(hfac)}^*}$, E_{hfac^*} and $E_{\mathrm{Pd(hfac)_2}^*}$ were the total energies of Pd(hfac)*, hfac* and $Pd(hfac)_2$* respectively.

The adsorption energy $\Delta E_{\mathrm{ads\text{-}species}}$ of $Pd(hfac)_2$, Pd(hfac) or hfac on Si (100) surface was calculated as equation (S3).

$$\Delta E_{\mathrm{ads\text{-}species}} = E_{\mathrm{species}^*} - E_{\mathrm{surface}} - E_{\mathrm{species(g)}} \quad \text{(S3)}$$

Where E_{species^*} was the total energy of $Pd(hfac)_2$*, Pd(hfac)* or hfac*. E_{species} represented the chemical potential of the $Pd(hfac)_2$, Pd(hfac) or hfac.

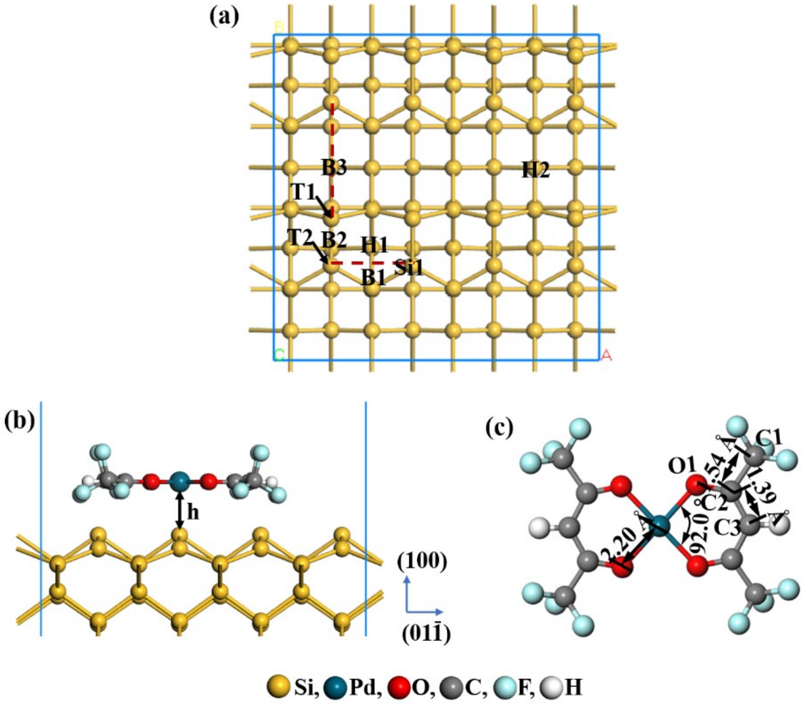

Fig. S3 Configurations of **(a)** bare Si (100) (top view), **(b)** bare Si (100) with Pd(hfac)$_2$ (side view) (h0 = 3.0 Å), and the structure of Pd(hfac)$_2$ precursor.

Since we focused on the reaction mechanism between ligand and ozone rather than the adsorption process, Si (100) slab instead of Al_2O_3 (100) slab was used to simplify the simulation process. A reconstructed Si (100) slab of seven layers with a vacuum space of 15 Å was used in this work, simulating the actual substrate used, which was consistent with other studies.[5,6] The bottom three substrate layers were kept fixed at their bulk positions to represent the infinitely large solid. Then, the other atomic layers were relaxed. Besides, seven different adsorption sites were considered, including top 1 (T1), top 2 (T2), bridge 1 (B1), bridge 2 (B2), bridge 3 (B3), hollow 1 (H1), and hollow 2 (H2) on bare Si (100) surface (Fig. S3a). Moreover, for each site, different adsorption orientations of species were carried out. In all initial structures, the height of the species was set at h_0 = 3 Å (Fig. S3b).

2.2 Results and Discussion

2.2.1 Chemisorption of Pd(hfac)$_2$ on bare Si (100) surface

The first step of a typical ALD process is the chemisorption of precursors at reactive sites, thus, resulting in the release of by-products. At first, the optimized structure of Pd(hfac)$_2$ was analyzed, and the results are shown in Fig. S3c. Pd coordinates four oxygens of two hfac ligands, and the length of the covalent Pd-O bonds is 2.00 Å. The

mean angle of O-Pd-O is 92.0°. Meanwhile, the bond lengths for C1-C2 and C2-C3 (marked in Fig. S3c) are 1.54 and 1.39 Å respectively. Those results are consistent with the experiment and DFT results reported by Siedle et al.[7] and Basova et al.[8] respectively (Table S1).

Table S1 Simulated and experimental bond lengths (Å) and angles (◦) of $Pd(hfac)_2$. The atomic labels are given in Fig. S3(c).

	**GGA-PW91**	**Experiment[7]**	**B3LYP[8]**
**Pd-O**	2.00	1.97	2.01
**O-Pd-O**	92.0	90.0	92.8
**C1-C2**	1.54	1.53	1.54
**C2-C3**	1.39	1.39	1.39

To elucidate the reaction mechanisms of $Pd(hfac)_2$ on bare Si (100) surface in detail, DFT calculation was employed. Additionally, Mulliken bond populations with 3.0 Å distance cut-off were carried out to evaluate the relative strength of the bonds. A high positive bond population value implies a strong bond strength. As seen in Fig. S4a, the most stable configuration for the adsorption of $Pd(hfac)_2$ on bare Si (100) surface is obtained at B2 site. The adsorption energy is -2.79 eV, indicating that $Pd(hfac)_2$ strongly chemisorbs on bare Si (100) surface. Moreover, the $Pd(hfac)_2$* is significantly distorted

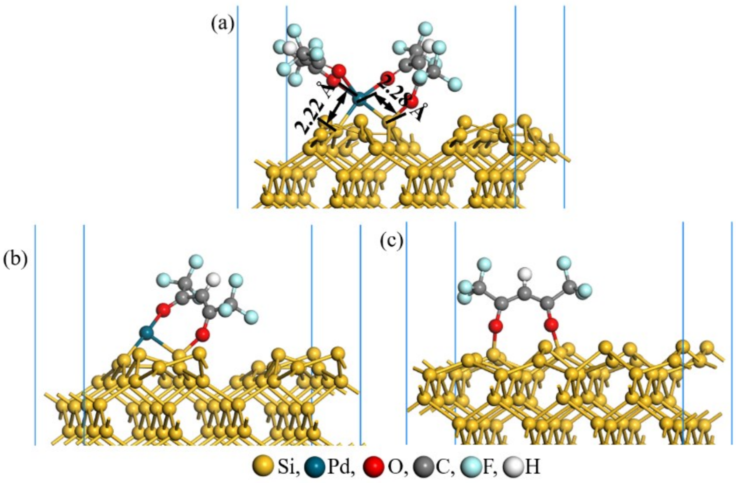

Fig. S4 Optimized geometries of different species (**(a)** $Pd(hfac)_2$*, **(b)** Pd(hfac)* and **(c)** hfac*) on clean Si (100) surface.

from planarity to improve the adsorption stability, associated with the dissociative adsorption of $Pd(hfac)_2$. Pd atom bonds with two neighboring Si atoms, leading to the efficient annihilation of the dangling bonds. The lengths of the Pd-Si bonds are 2.22 ~

2.28 Å, and the populations of the Pd-Si bonds are estimated to be 0.20 ~ 0.23, which imply that the formed Pd-Si bonds are strong. Also, the lengths of Pd-O bonds increase to 2.23 ~ 2.45 Å, indicating that the strength of Pd-O bonds is weakened.

The lowest reaction energy for reaction (S1) on bare Si (100) surface is -1.87 eV, which means that the reaction is possible. DFT calculations show that $Pd(hfac)_2$* dissociates to Pd(hfac)* and hfac*, whose optimized structures are shown in Fig. S4b and c. The lowest adsorption energy of Pd(hfac) on bare Si (100) surface is -4.08 eV. Two Pd-Si bonds are formed after Pd(hfac) adsorption at B2 site. The coordination configuration of Pd is preserved with a coordination number of 3, different from the gas phase $Pd(hfac)_2$ precursor. Fig. S4c shows the most stable structure of hfac adsorption on bare Si (100) surface. Hfac prefers to adsorb with the species axis being perpendicular at B1 site with the adsorption energy of -4.21 eV. The two carbonyl groups of hfac* adsorbs on two neighboring Si atoms.

2.2.2 Surface reaction between Pd(hfac) and ozone

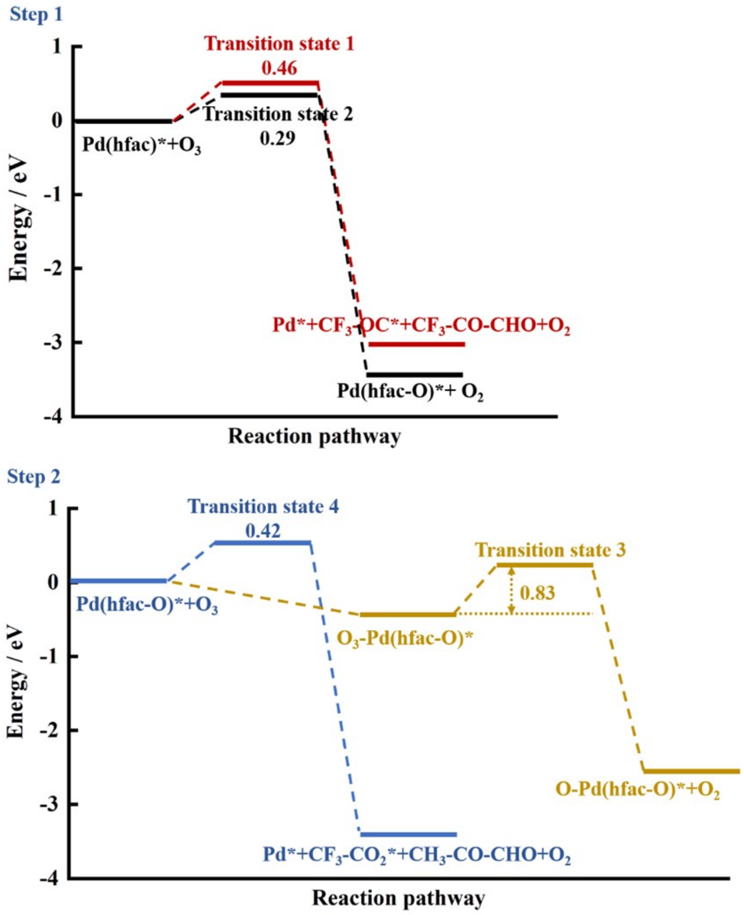

Fig. S5 The calculated potential energy profiles of the reactions between Pd(hfac)* and ozone (step 1) and between Pd(hfac-O)* and ozone (step 2) on clean Si (100) surface.

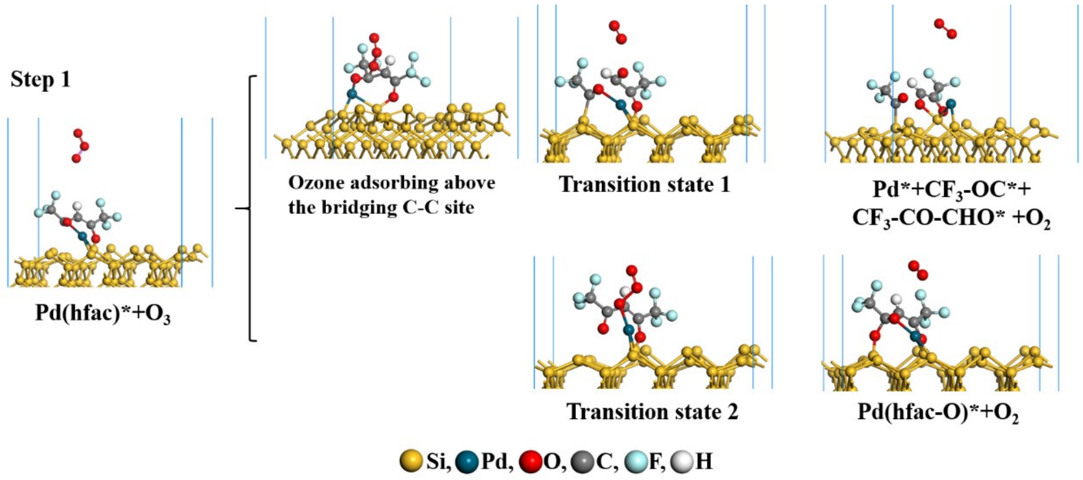

Fig. S6 Geometric structures for the reaction between Pd(hfac)* and ozone on clean Si (100) surface.

Since the ligands are used to volatilize the metal atoms, the ligands are expected to remain intact upon adsorption and removed by the co-reactant during the second step of the palladium ALD process. Because of Pd(hfac)$_2$* dissociation to form Pd(hfac)* and hfac*, as mentioned above, the reaction between Pd(hfac)* and ozone is studied. The potential energy profiles and optimized reactant, transition state, and product structures are shown in Fig. S5 and Fig. S6. As ozone approaches (step 1, Fig. S5), two reaction pathways are obtained. The activation barrier of the reaction between ozone and Pd(hfac)* is 0.46 eV. The relaxed adsorption structure with the adsorption energy of -3.01 eV is shown in Fig. S6. The reaction starts with an O atom of ozone adsorbing above the bridging C2-C3 site (as marked in Fig. S3c) to form an epoxide (the inset in Fig. S6). Subsequently, the adsorbed ozone molecule undergoes a dissociative chemisorption process. An O atom of the ozone molecule cleaves the C2-C3 bond, causing the O-C3 bond and oxygen molecule formation on the surface (the transition state 1 inset in Fig. S6). These results are consistent with the reaction between trimethylaluminum and ozone by DFT calculations and infrared spectroscopy.[9,10] Besides, as the C2-C3 bond is broken, the distance between the O1 (as marked in Fig. S3c) and Pd atoms of Pd(hfac)* increases from 2.12 Å to 4.09 Å, indicating that the O1-Pd bond is cleaved. As a result, the formed complex dissociates into the adsorbed Pd, CF_3-OC and CF_3-CO-CHO (cited as "Pd*", "CF_3-OC*" and "CF_3-CO-CHO*" respectively). According to the experiment results reported by Weber et al.,[11] gaseous fragments of the ligand including CF_3- might be formed, C atom, except for the C atom of -CF_3, might be oxidized into CO_2, and H atom might be oxidized to form H_2O.

A lower activation barrier of 0.29 eV is obtained by the reaction between Pd(hfac)* and ozone being at the top Pd site, implying the reaction is kinetically more favorable. An O atom of the ozone is reactive towards the Pd atom of Pd(hfac)*. Then, the distance between the O1 and the Pd atom increases from 2.12 Å to 4.50 Å, which implies that the O1-Pd bond is broken. Hence, the O1-Si bond is formed due to the transfer of the residual electrons on the O atom to the topmost Si atom. Besides, the adsorbed O atom of the ozone bonds with the C2 atom, causing the adsorbed Pd(hfac-O) (cited as "Pd(hfac-O)*") and oxygen formation. The adsorption energy is -3.46 eV.

As another ozone approaches the Pd(hfac-O)*, two reaction pathways are also found (step 2, Fig. S5), and the optimized reactant, transition state, and product structures are shown in Fig. S7. A stable structure with an activation barrier of 0.42 eV might be formed by the reaction between Pd(hfac)-O* and ozone at the bridging C-C site. The adsorption energy is -3.14 eV. The reaction process is shown in Fig. S5 (blue lines) and Fig. S7, which is similar to the reaction described in Fig. S5 (red lines) and Fig. S6. The final product is the Pd*, adsorbed CF_3-CO_2 and CF_3-CO-CHO*. The latter two might be oxidized to form CO_2, H_2O and gaseous fluoride.

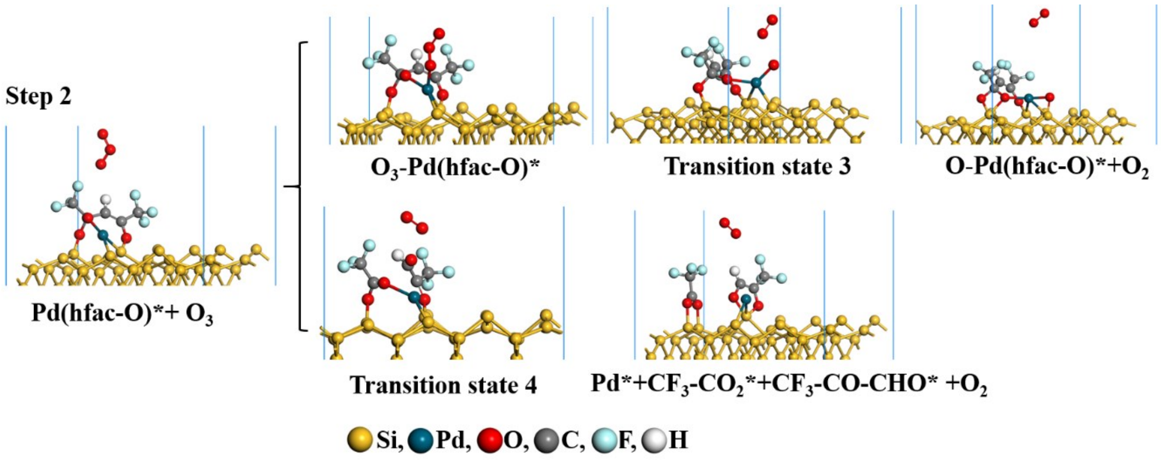

Fig. S7 Geometric structures for the reaction between Pd(hfac-O)* and ozone on clean Si (100) surface.

An O atom of the ozone might also adsorb barrierless at the top Pd site with the adsorption energy of -0.58 eV, resulting in O_3-Pd(hfac-O)* formation with no other bonds being broken, as displayed in Fig. S7. A decomposition of the O_3-Pd(hfac-O)* is then assumed (reaction (S4)). The reaction energy for reaction (S4) is written as: $\Delta E_{re4} = E_{\text{O-Pd(hfac-O)*}} + E_{O_2} - E_{O_3\text{-Pd(hfac-O)*}}$. The calculated reaction energy is -2.06 eV, revealing that further decomposition is favorable. The adsorbed O atom bonds with the

Pd and Si atoms to improve the structure stability. The activation barrier of the reaction is 0.83 eV.

$$O_3\text{-Pd(hfac-O)}^* \rightarrow \text{O-Pd(hfac-O)}^* + O_2 \quad \text{(S4)}$$

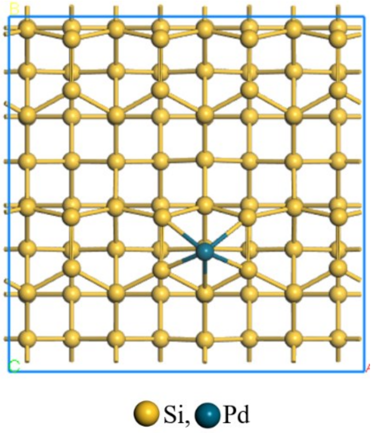

Fig. S8 Optimized geometry of Pd atom adsorption on clean Si (100) surface.

As the ligand is consumed, atomic Pd moves might occur. Fig. S8 depicts the most stable structure of atomic Pd adsorption on bare Si (100) surface. Pd atom prefers to adsorb at H1 site with the adsorption energy of -0.21 eV due to the most efficient annihilation of the dangling bonds in the topmost atoms.

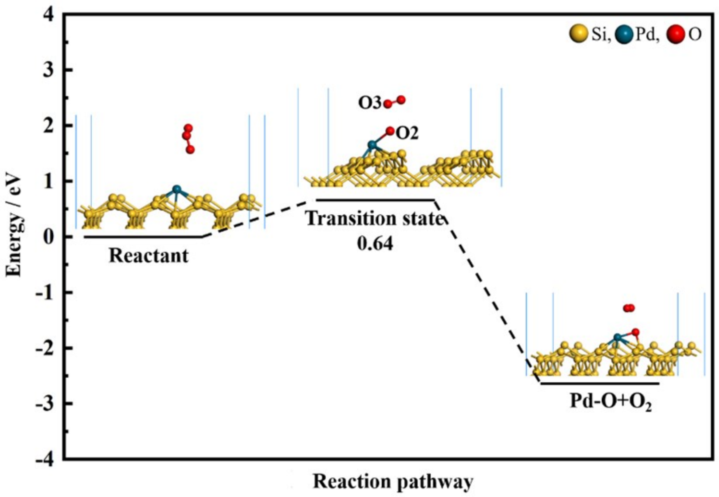

Fig. S9 The calculated potential energy profiles of the reaction between Pd* and ozone on clean Si (100) surface with the optimized reactant, transition state, and product structures.

When more ozone molecules are available, ozone might react with Pd* directly, as shown in Fig. S9. The activation barrier is 0.64 eV. An O atom of the ozone at the bridging Pd-Si site prefers to bond with the Pd and Si atoms to form Pd-O-Si bonds, resulting in the distance between the O_2 and O_3 atoms (marked in Fig. S9) increases

from 1.28 Å to 2.55 Å, which indicates that the O_2-O_3 bond is broken. The final adsorption energy of -2.64 eV.

In conclusion, palladium metal can be synthesized when the reaction condition is carefully controlled, especially the ozone concentration. Less ozone should be used to prepare palladium metal.

References

1 Z. Weng-Sieh, R. Gronsky and A. T. Bell, *J Catal*, 1997, **170**, 62-74.

2 H. J. Monkhorst and J. D. Pack, *Phys.Rev. B*, 1976, **13**, 5188.

3 G. Henkelman, B. P. Uberuaga and H. Jónsson, *J. Chem.Phys.*, 2000, **113**, 9901-9904.

4 G. Henkelman and H. Jónsson, *J. Chem.Phys.*, 2000, **113**, 9978-9985.

5 Y. Lei, B. Liu, J. Lu, X. Lin, L. Gao, N. P. Guisinger, J. P. Greeley and J. W. Elam, *Phys. Chem. Chem. Phys.*, 2015, **17**, 6470-6477.

6 M. Palummo, G. Onida, R. Del Sole and B. S. Mendoza, *Phys. Rev. B*, 1999, **60**, 2522.

7 X. Lu, Q. Zhang and M. Lin, *Phys. Chem. Chem. Phys.*, 2001, **3**, 2156-2161.

8 A. R. Siedle, R. A. Newmark and L. H. Pignolet, *Inorg. Chem.*, 1983, **22**, 2281-2286.

9 T. V. Basova, V. G. Kiselev, E. S. Filatov, L. A. Sheludyakova and I. K. Igumenov, *Vib. Spectrosc.*, 2012, **61**, 219-225.

10 D. N. Goldstein, J. A. McCormick and S. M. George, *J. Phys. Chem. C*, 2008, **112**, 19530-19539.

11 S. D. Elliott, G. Scarel, C. Wiemer, M. Fanciulli and G. Pavia, *Chem. Mater.*, 2006, **18**, 3764-3773.

12 M. J. Weber, A. J. Mackus, M. A. Verheijen, V. Longo, A. A. Bol and W. M. Kessels, *J. Phys. Chem. C*, 2014, **118**, 8702-8711.
